# Analysis of Predictive Factors for Return to Sports in Female Athletes With Stress Urinary Incontinence

**DOI:** 10.7759/cureus.44364

**Published:** 2023-08-30

**Authors:** Nobuo Okui, Tamer Erel, Machiko Aurora Okui

**Affiliations:** 1 Dentistry, Kanagawa Dental University, Yokosuka, JPN; 2 Department of Obstetrics and Gynecology, Istanbul University, Cerrahpasa School of Medicine, Istanbul, TUR; 3 Department of Urogynecology, Yokosuka Urogynecology Clinic, Yokosuka, JPN

**Keywords:** ai and robotics in healthcare, urethral erbium laser for urinary incontinence, vaginal erbium laser for urinary incontinance, chatgpt, serum total testosterone, pelvic floor muscle training, stress urinary incontinence, elite female athletes

## Abstract

Introduction

This study aimed to identify predictive factors for successful return to sports among elite female athletes (EFAs) experiencing stress urinary incontinence (SUI). We used machine learning to analyze these predictors.

Methods

This study was conducted at Yokosuka Urogynecology and Urology Clinic, located in Yokosuka City, Kanagawa, Japan. A total of 153 EFAs with postpartum SUI were included in this retrospective cohort study. Information regarding the frequency of pelvic floor muscle training (PFMT), treatment approaches, rates of return to sports after one year, and one-hour pad test (1HrPadtest) at three months were collected.

Results

At three months, 26.8% of the EFAs improved in SUI; after one year, 28.1% returned to their respective sports successfully. The equation for predicting return to sports (logit(p)) involved several factors: (a) serum total testosterone, (b) PFMT frequency per week, (c) 1HrPadtest at three months, and (d) vaginal erbium-doped yttrium aluminum garnet laser (VEL) + urethral EL (UEL) treatment. The equation was as follows: -126 - 0.07276a + 25.98b - 1.947c - 25.32d, with a logit(p) cutoff point at 0.5. The optimal cutoff values and the four influential factors were determined through a receiver operating characteristic (ROC) analysis and the random forest model, respectively.

Conclusions

For EFAs with severe SUI to successfully return to their sports activities, the PFMT frequency was paramount. Patients who exhibited unsatisfactory results in the 1HrPadtest at the three-month mark benefited from the VEL+UEL treatment. Serum total testosterone proved to be an effective discerning criterion.

## Introduction

Stress urinary incontinence (SUI), especially in the postpartum period, poses a significant concern for elite female athletes (EFAs), potentially impairing their sports performance and quality of life [1-3]. To our knowledge, only one instance of a successful return to sport has been reported [1], leaving the current understanding of SUI among athletes and the efficacy of treatment methods limited. Notably, elite athletes facing severe postpartum SUI may encounter specific challenges when seeking to resume competitive sports.

Various treatment strategies exist for SUI. Among them, pelvic floor muscle training (PFMT) has been proven effective in managing SUI in EFAs [4,5]. Nevertheless, the essential predictive factors enabling successful sports reentry for EFAs with severe SUI, incorporating interventions, such as PFMT, remain unexplored. While we understand that there are limited published studies on the effectiveness of PFMT, specifically in EFAs [5], we recognize the importance of considering all existing research in this area. In addition to PFMT, other intervention strategies encompass therapeutic approaches, such as vaginal erbium-doped yttrium aluminum garnet (Er:YAG) laser (VEL) and urethral Er:YAG laser (UEL) [1], and surgical procedures, including mid-urethral sling (MUS) surgery [6].

However, the effectiveness of these interventions and their correlation with sports reentry in EFAs have not been investigated, indicating the need for statistical analysis in future treatments. This necessitates the development of methods to evaluate complex predictive factors. Recent technological advancements have fueled interest in applying artificial intelligence (AI) to medical statistics [7,8]. By using the OpenAI chat Generative Pre-trained Transformer 4 (ChatGPT, OpenAI, San Francisco, USA), which is capable of developing appropriate statistical programming, we can analyze intricate factors in our study and incorporate suitable approaches into our research.

The objective of this study was to identify the essential predictive factors for achieving a successful return to sports among EFAs with severe SUI. Our investigation included interventions, such as PFMT, VEL, UEL, and MUS. We evaluated the return-to-sport outcomes at the one-year mark and achieved a 2 g or lower score on the one-hour pad test (1HrPadtest) within three months.

## Materials and methods

Design

This is a retrospective cohort study of EFAs who visited Yokosuka Urogynecology and Urology Clinic, located in Yokosuka City, Kanagawa, Japan. The study was approved by the Regional Medical Ethics Committee (Ethical Review Board of Kanagawa Association of Medical and Dental Practitioners, with approval number 22003) and was registered at University Hospital Medical Information Network Clinical Trials Registry (UMIN-CTR) as R000057696.

Patients

In this retrospective cohort study, we included EFAs who had participated in long-distance marathons but could not continue participating in sports for more than six months owing to severe SUI and who visited our hospital from April 2015 to March 2020. The inclusion criteria were as follows: EFAs who competed under the elite category of the Athletics Federation. Accordingly, SUI was established. Then, the participants underwent an assessment of normal cardiopulmonary and coagulation function, facilitated by electrocardiogram measurements and blood tests. We did not differentiate between vaginal delivery and cesarean section. The exclusion criteria were as follows: uterine and ovarian diseases, breast cancer, neurogenic bladder, congenital anomalies of the urethra, and ureteral orifice malformations. Patients with mixed urinary incontinence, urgency urinary incontinence, dysfunctional voiding, and other lower urinary tract symptoms were excluded. Patients who did not undergo post-treatment assessments or did not provide consent were also excluded from the study.

Study parameters

Clinical data on the following parameters were collected: age, pre-pregnancy history of urinary leakage, number of childbirths, mode of delivery (vaginal delivery or cesarean section), number of training days per week, duration of training (years), history of cardiac disease/arrhythmia, angina, asthma, chronic bronchitis, fractures or joint injuries, history of previous pelvic surgery, diabetes, thyroid dysfunction, menstrual abnormalities or amenorrhea, relative energy deficiency in sports [9], depression, bladder neck descent (cm), levator hiatus distensibility and contractility measured using ultrasound [10], testosterone levels (resting and early morning), duration of daily PFMT, frequency of PFMT per week, International Consultation on Incontinence Questionnaire-Urinary Incontinence Short Form (ICIQ-UI SF) score [1], 1HrPadtest score [1], VEL + UEL [10], MUS surgery [11], 1HrPadtest score at three months after treatment, and return-to-sport rate at one year.

Blood sampling was collected at 9 a.m. during the ovulation phase. Ovulation was confirmed in each EFA using ultrasonography and the analysis of temperature charts submitted by the patients. In addition, it was confirmed that EFAs did not use testosterone to enhance their performance. The following parameters were measured at Bio Medical Laboratories, Inc., Tokyo, Japan: total testosterone level, as measured using chemiluminescence immunoassay method (normal range: 10.8-56.9 ng/dL); estradiol level, as measured using chemiluminescence immunoassay method (normal range: 49-487 pg/mL); total cholesterol level [12], as measured using an enzymatic method (normal range: 150-219 mg/dL); hemoglobin level [13], as measured using the sodium lauryl sulphate-hemoglobin (SLS-Hb) method (normal range: 11.2-15.2 g/dL); and urea nitrogen level [14], as measured using the urease-glutamate dehydrogenase-ultraviolet method (normal range: 8.0-20.0 mg/dL).

Treatment schedule

During the initial consultation, we verified the presence of SUI that had been negatively impacting sports activities for over six months. It was a foundational tenet of our treatment protocol to minimize the duration during which EFAs could not participate in sports. Specifically, for all EFAs, we emphasized PFMT as the primary option for the first month. Within the initial month following the first visit, we inquired about the interest in additional treatments. Regardless of the chosen additional treatments, PFMT was administered for 16 weeks. For all EFAs, the 1HrPadtest score three months after the final session of PFMT and the return-to-sport outcomes at one year were evaluated. Return to sports after one year was determined through self-reporting. If individuals regarded their performance as below the "elite" level, it was deemed that they had not fully returned to sports.

PFMT

Conducted uniformly for all EFAs, PFMT was carried out for 16 weeks. During the initial visit, two physicians independently performed vaginal palpation. According to the definition by Okui et al. [1], PFM contractions were classified into invisible contractions, simultaneous contractions of auxiliary muscles, reverse contractions (Valsalva or tension manipulation), or correct PFM contractions. Subsequently, the opinions of the two physicians were integrated, and the participants were instructed on appropriate PFM contractions. YouTube videos were created to facilitate ongoing PFMT at home. The participants recorded their PFMT sessions in a diary. The progress of PFMT was reviewed during monthly follow-up visits based on the diary entries.

The exercise regimen was implemented according to the designated protocol. This regimen consisted of three sets of 10 repetitions each of PFM contractions, with each contraction lasting between six and eight seconds. The intensity of these contractions was set at 60-70% of the individual's maximum voluntary contraction (MVC). In addition, two sets of 10 repetitions of PFM contractions were performed at an intensity ranging from 30 to 60% of the MVC. All contractions were synchronized with exhalations and concurrent contractions of the transversus abdominis muscle to ensure proper coordination. These exercises were conducted while lying on one's back with the legs bent and feet on the ground. Furthermore, the participants were advised to adopt the "Knack maneuver as a practical strategy to manage scenarios involving heightened intra-abdominal pressure during activities such as coughing, sneezing, laughing, or lifting heavy objects."

Considering their training as athletes, it was decided that individuals could modify the number of sets for PFMT.

Informed consent for additional treatments to PFMT

Additional interventions to PFMT were chosen by the participants themselves at the one-month point of PFMT. The options included the following: (a) urethral collagen injection [15], (b) MUS surgery, (c) open surgery [16], and (d) laser treatment [1]. Urethral collagen injection required overseas travel because of the unavailability of materials at the patients' residences. MUS surgery included tension-free vaginal tape and transobturator tape procedures using synthetic mesh tapes inserted through the urethra. These options were covered by national health insurance. Open surgery proposed fascial slings. The VEL+UEL treatment was a self-pay option. The benefits and risks of each treatment were thoroughly explained to the patients, and the final treatment selection was collaboratively determined with the patient's physician, thereby ensuring informed consent. In our study, no EFAs desired urethral collagen injection or open surgery; therefore, the protocol did not include any of the two options.

VEL+UEL treatment

Local anesthesia in the form of 8% lidocaine spray (Sandoz KK, Tokyo, Japan) was administered to the patient's urethra, labia, and vagina; however, no sedatives were used [1]. A specialized glass vaginal probe was used for the VEL treatment, performed with an IncontiLase protocol (SP Dynamis Fotona, Ljubljana, Slovenia). The anterior vaginal wall was treated with a PS03 laser probe at a frequency of 2.0 Hz, pulse energy of 6 J/cm^2^, and spot size of 7 mm. The area was targeted every 5 mm, and the procedure was repeated thrice. The UEL treatment used the R09-2Gu laser handpiece to irradiate the urethral canal at a frequency of 1.6 Hz and pulse energy density of 1.4 J/cm^2^. The laser was applied every 2.5 mm, and four passes were applied. The consecutive procedures of the VEL, UEL, and VEL+UEL treatments were completed in approximately 20 min [1].

MUS surgery

MUS surgery was performed as an outpatient procedure using the tension-free vaginal tape and transobturator tape approaches under lumbar anesthesia [6].

Statistical analysis

Continuous variables are reported as mean ± standard deviation or number (percentage). The Kruskal-Wallis test was used to compare the three groups. To ensure the validity of our analysis, we first assessed the normal distribution of the data. After confirming the normal distribution, we employed Pearson's correlation coefficient to evaluate the relationship between each parameter and the return-to-sport rate at one year. A multivariate backward stepwise logistic regression analysis [17] was conducted, including variables with a p-value < 0.05 in the univariate analysis. Receiver operating characteristic (ROC) curve analysis was performed to determine the optimal cutoff value for predicting the return-to-sport rate at one year. A random forest model was employed to further explore the significance of variables in predicting the return-to-sport rates in EFAs. The model was constructed with 100 trees, which assessed the variable importance based on the IncNodePurity measure. Statistical significance was defined as p ≤ 0.05. All analyses and selection of the study parameters were conducted using R statistical software version 2.15.1 (R Core Team, Austria, Vienna). The statistical methods and R programming were implemented using the ChatGPT-4, running on the Windows 10 version 1903 operating system (Microsoft Corporation, USA).

## Results

Overall, 168 EFAs with SUI after childbirth visited our clinic. The study group comprised women who had not reached menopause. Seven women who registered but did not attend follow-up visits and eight who discontinued the PFMT protocol or did not undergo the 1HrPadtest at three months were excluded. Finally, 153 EFAs were included as participants in the study. The average age of the participants was 38.2 ± 3.9 years, with an average of 1.93 ± 0.40 childbirth experiences. In addition, nine individuals reported pre-existing urinary incontinence before childbirth. Based on our analysis, the compliance rate with PFMT implementation records for the program was reported to be 92.4% ± 13.0.

No women from the VEL+UEL treatment or MUS surgery category withdrew from the study. At the three-month follow-up, SUI improvement was observed in 41 women (26.8%): 10 in the PFMT alone group, 27 in the PFMT/VEL+UEL treatment group, and four in the PFMT/MUS surgery group. At the one-year follow-up, successful return to sports was observed in 16 patients from the PFMT group, 27 from the PFMT/VEL+UEL group, and no patients from the PFMT/MUS surgery group.

To address concerns regarding potential selection bias in the additional treatment group, we conducted a rigorous comparison of baseline characteristics (Table 1). Comparisons between the three groups (PFMT group, PFMT/VEL+UEL group, and PFMT/MUS group) were performed using the Kruskal-Wallis test. Various factors, including age, pre-childbirth SUI, number of vaginal deliveries, and weekly training sessions, were compared between groups to assess the severity of urinary incontinence. In addition, physiological indicators, such as bladder neck descent, were examined. Analysis of pre-treatment one-hour pad test results revealed no statistically significant differences between the treatment groups (p = 0.889), indicating that the additional treatment group did not inherently have greater severity of urinary incontinence. Furthermore, the evaluation of the ICIQ-SF yielded similar results between treatment groups (p = 0.150), reinforcing the notion of limited bias influence.

**Table 1:** Demographics and populations of the three treatment groups Three groups: PFMT group, PFMT only; PFMT/MUS group, PFMT with MUS; PFMT/VEL+UEL group, PFMT with VEL+UEL. *The Kruskal-Wallis test was used to compare the three groups. PFMT, pelvic floor muscle training; VEL+UEL, vaginal erbium-doped yttrium aluminum garnet laser and urethral erbium-doped yttrium aluminum garnet laser; MUS, mid-urethral sling; SUI, stress urinary incontinence; ICIQ-SF, incontinence questionnaire-urinary incontinence short form; RED-S, relative energy deficiency in sports; BND, bladder neck descent; 1HrPadtest, one-hour pad test

Group	PFMT	PFMT/MUS	PFMT/VEL+UEL	p value*
Number	122	4	27	
Age (years)	38.28 ± 3.59	34.75 ± 4.09	38.33 ± 5.35	0.205
SUI before childbirth (%)	5	25	7	0.230
Number of vaginal deliveries	1.92 ± 0.40	1.50 ± 0.50	2.00 ± 0.38	0.419
Number of training sessions per week	6.09 ± 1.01	7.00 ± 0.00	5.96 ± 1.17	0.127
Cardiac disease (%)	0	0	4	0.097
Asthma (%)	2	0	4	0.753
Menstrual abnormalities/amenorrhea (%)	3	0	4	0.928
RED-S (%)	3	0	4	0.928
Uterine fibroids and endometriosis (%)	8	0	15	0.457
Ovarian cysts (%)	3	0	4	0.928
BND: cm	1.76 ± 0.08	1.85 ± 0.05	1.81 ± 0.08	0.002
Distensibility of the levator hiatus	17.24 ± 0.59	17.13 ± 0.74	17.20 ± 0.63	0.779
Contractility of the levator hiatus	12.58 ± 0.49	12.38 ± 0.22	12.70 ± 0.50	0.471
Total testosterone (morning, ovulation period): ng/dL	31.79 ± 8.49	28.0 ± 5.34	54.48 ± 5.87	0.001
Estradiol (morning, ovulation period): pg/mL	187.18 ± 74.21	273.50 ± 77.79	168.26 ± 80.98	0.0639
Total cholesterol: mg/dL	164.68 ± 21.82	170.50 ± 15.85	165.07 ± 25.59	0.852
Hemoglobin: g/dL	12.83 ± 0.55	13.80 ± 0.78	13.13 ± 0.61	0.012
Urea nitrogen: mg/dL	13.34 ± 1.87	13.78 ± 1.45	13.19 ± 1.71	0.551
ICIQ-SF	12.82 ± 2.12	13.00 ± 0.00	13.67 ± 0.94	0.150
1HrPadtest before treatment	70.89 ± 20.08	69.25 ± 16.21	76.93 ± 15.08	0.889

The approach suggested by ChatGPT-4 was to use Pearson's correlation and logistic multivariate analysis.

Table 2 shows Pearson's correlation coefficients (95% confidence intervals (CIs)) and corresponding p-values. A significant correlation was observed between the frequency of PFMT per week for the 1HrPadtest, ICIQ-SF score, presence of PFMT, presence of the VEL+UEL treatment, and number of individuals achieving continence in the 1HrPadtest at three months. Specifically, a strong correlation was observed between the number of individuals who became continent in the 1HrPadtest, the number of individuals who became continent with PFMT alone, and the number of individuals who became continent with the VEL+UEL treatment.

**Table 2:** Baseline characteristics and Pearson’s correlation coefficients Correlation coefficients (95% confidence intervals) and p-values for various variables associated with return-to-sport rates at one year and one-hour pad test (≤2 g) at three months before and after an intervention. 1HrPadtest, 1-hour pad test; SUI, stress urinary incontinence; ICIQ-SF, International Consultation on Incontinence Questionnaire–Urinary Incontinence Short Form; PFMT, pelvic floor muscle training; VEL+UEL, vaginal erbium-doped yttrium aluminum garnet laser and urethral erbium-doped yttrium aluminum garnet laser; MUS, mid-urethral sling

Variable	Values	Return to sport at one year		1HrPadtest ≤ 2 g at three months	
		Correlation coefficient (95% confidence interval)	P	Correlation coefficient (95% confidence interval)	P
Before treatment					
Age (years)	38.2 ± 14.8	-0.0697 (-0.226-0.09)	0.392	-0.00518 (-0.164-0.154)	0.949
SUI before childbirth	9 (5.9)	-0.0327 (-0.19-0.127)	0.688	0.0369 (-0.123-0.194)	0.651
Number of vaginal deliveries	1.93 ± 0.23	-0.0332 (-0.191-0.126)	0.681	-0.00194 (-0.161-0.157)	0.981
Cesarean section	—	—	—	—	—
Number of training sessions per week	6.09 ± 1.04	0.000916 (-0.158-0.16)	0.991	-0.0249 (-0.183-0.134)	0.76
Cardiac disease	1 (0.65)	—	—	—	—
Asthma	3(1.96)	0.0165 (-0.143-0.175)	0.84	0.0209 (-0.138-0.179)	0.798
Fracture/arthritis	—	—	—	—	—
Thyroid disease	—	—	—	—	—
Diabetes	—	—	—	—	—
Previous pelvic surgery	—	—	—	—	—
Menstrual abnormalities/amenorrhea	5(3.26)	-0.0331 (-0.191-0.126)	0.684	-0.0282 (-0.186-0.131)	0.729
RED-S: relative energy deficiency in sports	5(3.26)	-0.0331 (-0.191-0.126)	0.684	-0.0282 (-0.186-0.131)	0.729
Depression	—	—	—	—	—
Uterine fibroids and endometriosis	14(9.2)	2.86(0.94-8.72)	0.0644	0.115 (-0.0444-0.269)	0.157
Ovarian cysts	5(3.26)	-0.0331 (-0.191-0.126)	0.684	-0.0282 (-0.186-0.131)	0.729
Bladder neck descent (BND): cm	1.77 ± 0.085	0.157 (-0.308--0.00133)	0.052	-0.203 (-0.35--0.0453)	0.012
Distensibility of the levator hiatus	17.22 ± 0.60	0.0492 (-0.11-0.206)	0.546	0.0409 (-0.119-0.198)	0.616
Contractility of the levator hiatus	12.6 ± 0.49	0.0914 (-0.0682-0.247)	0.261	0.0428 (-0.117-0.2)	0.599
Total testosterone (morning, ovulation period): ng/dL	35.7 ± 11.83	0.74 (0.659-0.804)	<0.001	0.928 (0.902-0.947)	<0.001
Estradiol (morning, ovulation period): pg/mL	185.5± 76.7	0.056 (-0.103-0.213)	0.486	-0.045 (-0.202-0.114)	0.58
Total cholesterol: mg/dL	164.8± 22.4	0.079 (-0.0807-0.235)	0.332	-0.112 (-0.266-0.0477)	0.169
Hemoglobin: g/dL	12.9 ± 0.57	0.104 (-0.0552-0.259)	0.199	-0.135 (-0.287-0.0244)	0.0967
Urea nitrogen: mg/dL	13.3 ± 1.83	-0.0208 (-0.179-0.138)	0.799	-0.009 (-0.168-0.149)	0.903
ICIQ-SF	12.97± 1.96	-0.243 (-0.387--0.0882)	0.002	-0.263 (-0.404--0.108)	0.001
1HrPadtest before treatment	72.07± 19.3	-0.313 (-0.45--0.163)	0.002	-0.246 (-0.39--0.091)	0.002
Intervention					
PFMT per day (hour)	1.04 ± 0.379	0.045 (-0.114-0.202)	0.581	-0.0678 (-0.224-0.0918)	0.405
Frequency of PFMT per week	6.11± 0.567	0.75 (0.671-0.812)	<0.001	0.611 (0.5-0.701)	<0.001
VEL+UEL treatment	27(17.6)	0.74 (0.659-0.805)	<0.001	0.765 (0.69-0.824)	<0.001
MUS surgery	4(2.6)	-0.102 (-0.257-0.0572)	0.208	0.271 (0.117-0.412)	<0.001
3 months after treatment					
1HrPadtest at 3 months after treatment	34.3± 25.0	-0.808 (-0.857--0.744)	<0.001	—	—
Resolution of urinary incontinence (1HrPadtest ≤ 2g) at 3 months after treatment.	41(26.8)	0.804 (0.739-0.853)	<0.001	—	—
Resolution of urinary incontinence (1HrPadtest ≤ 2g) at 3 months after PFMT only	10 (8.2)	-0.755 (-0.821--0.667)	<0.001	—	—
Resolution of urinary incontinence (1HrPadtest ≤ 2g) at 3 months after VEL+UEL treatment	27 (100)	0.74 (0.659-0.805)	<0.001	—	—
Resolution of urinary incontinence (1HrPadtest ≤ 2g) at 3 months after MUS surgery	4 (100)	-0.102 (-0.257-0.0572)	0.208	—	—
1 year after treatment					
Return to sports at 1 year	43 (28.1)	—	—		

Correlation analysis revealed a significant positive correlation between the VEL+UEL treatment and return-to-sport rates (r = 0.74, 95% CI: 0.659-0.805, p < 0.001). In addition, a strong positive correlation was observed between the number of PFMT sessions per week and return-to-sport rates (r = 0.75, 95% CI: 0.671-0.812, p < 0.001). Conversely, a negative correlation was noted between the 1HrPadtest results at three months and return-to-sport rates (r = -0.808, 95% CI: -0.857 to -0.744, p < 0.001).

Multivariate analysis, which was performed to examine the association between explanatory variables and returned rates in female athletes with SUI using logistic regression, revealed a significant positive association between the serum total testosterone levels and return rates (odds ratio (OR): 1.011, 95% CI: 1.005-1.017, p < 0.001). In addition, a significant positive association was noted between the frequency of PFMT per week and return rates (OR: 1.319, 95% CI: 1.205-1.444, p < 0.001). Conversely, the 1HrPadtest at three months demonstrated a significant negative association with return rates (OR: 0.993, 95% CI: 0.995-0.997, p < 0.001). The variable VEL.UEL demonstrated a significant predictor of return rates in female athletes with SUI. The OR for VEL.UEL was 1.863 (95% CI: 1.583-2.193, p < 0.001), further supporting its significant impact on return rates.

The ChatGPT-4 selected the following four factors for logit(p) calculation: (a) serum total testosterone, (b) frequency of PFMT per week, (c) 1HrPadtest at three months, and (d) VEL+UEL. The predicted logit (p) can be expressed as follows: logit(p) = -126 - 0.07276a + 25.98b - 1.947c - 25.32d. The mean and standard deviation of logit(p) were -41.21 and 53.52, respectively.

**Figure 1:**
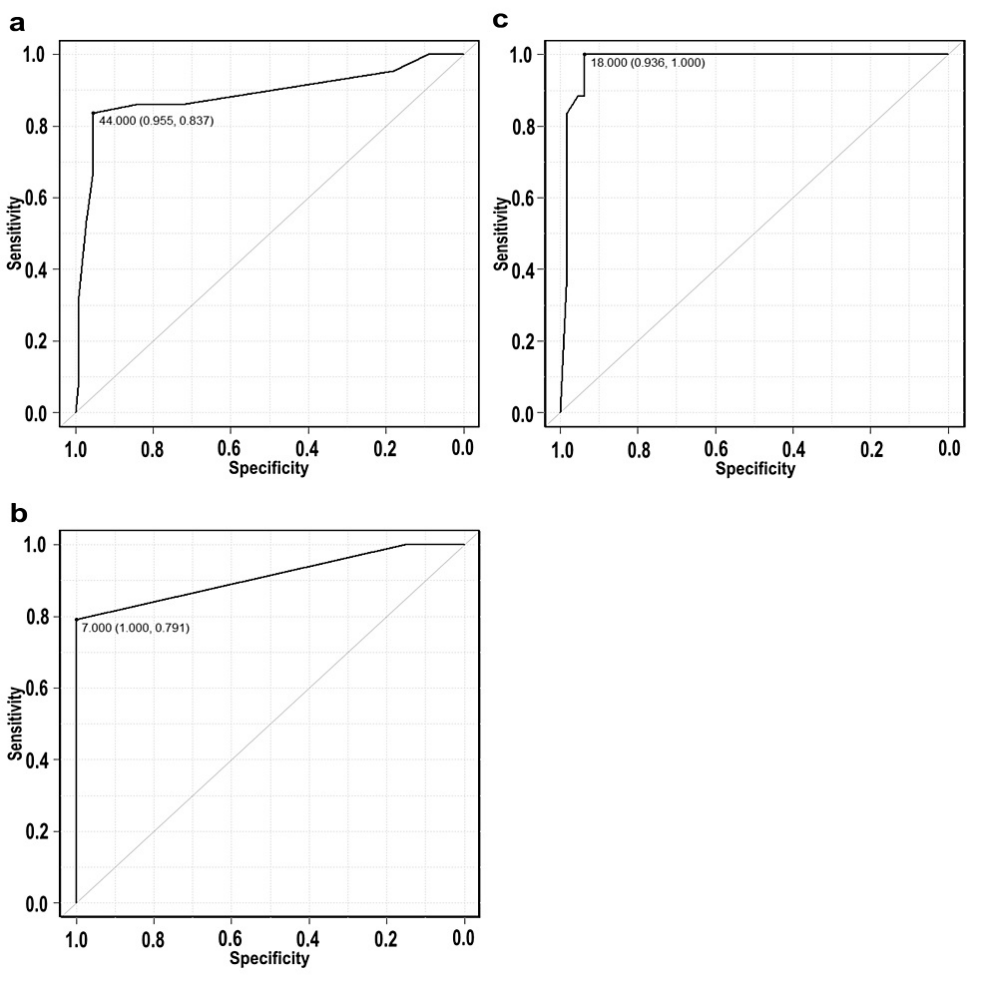
ROC analysis and cutoff values (a) ROC curve for the relationship between serum total testosterone and return-to-sport rates. (b) ROC curve for the cutoff value of PFMT frequency per week and return-to-sport rates. (c) ROC curve for the cutoff value of the 1HrPadtest at three months and return-to-sport rates. In each figure, specificity is plotted on the X-axis ranging from 1.0 to 0.0, and sensitivity is plotted on the Y-axis ranging from 0.0 to 1.0. The AUC (confidence interval) for Figure 1a is 44.000 (0.955, 0.837), for Figure 1b is 7.000 (1.000, 0.791), and for Figure 1c is 18.000 (0.936, 1.000). 1HrPadtest: 1-hour pad test, AUC: area under the curve, PFMT: pelvic floor muscle training, ROC: receiver operating characteristic

The ChatGPT-4 performed ROC analysis and selected the following factors to determine optimal cutoff values: PFMT frequency per week, total testosterone levels, and 1HrPadtest at three months. The area under the curve (AUC) for the total testosterone levels and return-to-sport rates at one year was 0.888 (95% CI: 0.816-0.96) (Figure 1a). The AUC for the cutoff value of the PFMT frequency per week and return-to-sport rates was 0.912 (95% CI: 0.859-0.964) (Figure 1b). In addition, the AUC for the cutoff value of the 1HrPadtest at three months and return-to-sport rates was 0.979 (95% CI: 0.958-1.0) (Figure 1c).

ROC curves were generated to predict the return rates at one year using the following factors: (a) total testosterone levels, (b) frequency of PFMT per week, and (c) 1HrPadtest at three months.

Other factors, including MUS surgery, duration of PFMT per day, duration of training, number of training days per week, number of childbirths, and pre-pregnancy history of urinary leakage, did not show significant correlations with return-to-sport rates.

In addition to the correlation and logistic regression analyses, a random forest model proposed by ChatGPT was employed to further investigate the importance of variables in predicting the return-to-sport rates in EFAs. The random forest model used the return rates at one year as the target variable. By contrast, the explanatory variables included the 1HrPadtest at three months, total testosterone levels, VEL+UEL treatment, and frequency of PFMT per week. The dataset was classified into a training set (70% of the data) and a test set (30% of the data).

**Figure 2:**
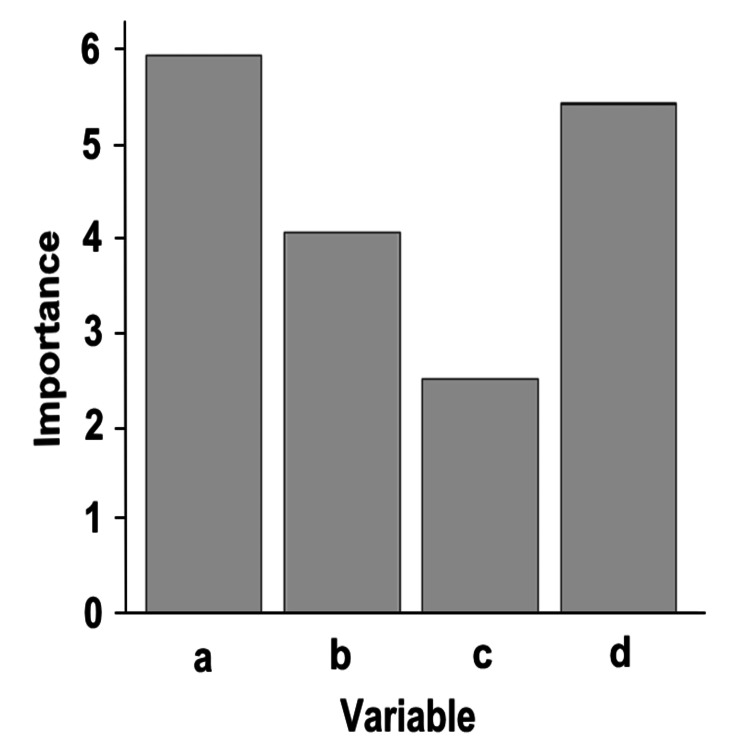
Random forest variable importance The vertical axis represents the importance of variables when the target variable is set as "return rates at one year" in the random forest model. The horizontal axis represents the variables, with the following corresponding labels: (a) 1HrPadtest at three months, (b) total testosterone levels, (c) VEL+UEL treatment, and (d) frequency of PFMT per week. 1HrPadtest: 1-hour pad test, PFMT: pelvic floor muscle training, VEL+UEL: vaginal erbium-doped yttrium aluminum garnet laser and urethral erbium-doped yttrium aluminum garnet laser

The random forest model provided valuable insights into the importance of these variables in predicting the return-to-sport rates (Figure 2). The variable importance values were calculated, and the results indicated that the 1HrPadtest at three months had the highest importance value of 5.945126, followed by the frequency of PFMT per week with an importance value of 5.437675. The total testosterone levels and VEL+UEL treatment also demonstrated significant importance, with values of 4.059365 and 2.483492, respectively. These importance values indicate the relative contribution of each variable to the prediction of return-to-sport rates, suggesting that the 1HrPadtest at three months and frequency of PFMT per week are crucial factors for predicting the athletes' ability to return to sports.

## Discussion

In our study, we focused on PFMT as the intervention method, which is known to be effective in managing SUI in EFAs [18]. Our findings provide valuable insights into the relationship between predictive factors and successful return rates in EFAs with severe SUI. The strongest predictor of return rates after one year was determined to be the 1HrPadtest conducted at three months after interventions. Individuals with a lower 1HrPadtest value at three months had a higher likelihood of returning to sports. This underscores the importance of timely and effective interventions to address SUI symptoms, as early resolution of urinary incontinence appears to be associated with better chances of returning to sports.

A significant positive correlation was observed between the frequency of PFMT per week and return-to-sport rates, indicating that a higher frequency of PFMT was associated with higher rates of return. This finding aligns with previous research that highlights the effectiveness of PFMT in improving PFM strength and control [3-5,18-20]. Regular and consistent PFMT sessions likely restore urinary continence and subsequent return to sports activities [3,18-20]. Furthermore, studies have reported the impact of pelvic floor symptoms on women’s participation in sports and exercise, emphasizing the need for strategies to address and manage these symptoms [18,20]. To the best of our knowledge, no previous studies have reported on the effects of PFMT on the return of EFAs, making these findings particularly valuable.

The presence of the VEL+UEL treatment demonstrated a significant positive correlation with return-to-sport rates. The VEL+UEL treatment has been proposed as a promising intervention for SUI [1,21]. The positive correlation observed in our study indicates that implementing the VEL+UEL treatment contributes to improved outcomes and higher rates of return among EFAs. This finding aligns with the findings of previous research highlighting the efficacy of concomitant VEL+UEL laser treatment in improving SUI symptoms, especially in postmenopausal women [21]. The mechanism of the VEL+UEL treatment is believed to enhance the support of PFM, as demonstrated by MRI [1]. Adding UEL to VEL is predicted to provide even greater support and effectiveness [21]. Therefore, the VEL+UEL treatment may be a suitable intervention for individuals with unsatisfactory 1HrPadtest results after three months of PFMT.

Furthermore, we conducted an ROC analysis to determine the optimal cutoff values for total testosterone levels, frequency of PFMT per week, and 1HrPadtest at three months as predictors of return-to-sport rates [18,20-22]. The AUC for these variables were 0.888, 0.912, and 0.979, respectively [18,22-24]. These results indicate the potential use of these variables in predicting the likelihood of successful return to sports among EFAs with SUI [18,22-24]. In addition, previous studies have demonstrated that low serum testosterone levels are associated with an increased likelihood of stress and mixed incontinence in women [18,22,23]. Furthermore, testosterone administration has increased muscle mass, including the trunk and pelvis core muscles [18,22,24]. These findings indicate a possible relationship between testosterone levels, muscle strength, and their potential influence on SUI and the ability to return to sports [18,22-24]. Regarding the correlation between serum testosterone levels and return to sports at one year, a testosterone peak during ovulation indicated that higher levels of serum total testosterone are independently associated with an increased likelihood of return to sports of female athletes after childbirth. However, this association should be further investigated after controlling for other variables.

The disparity between the frequency of PFMT sessions per week and the duration of PFMT per day stems from the multifaceted nature of PMFT as an intervention approach. Because of this rationale, certain factors, such as the weekly frequency of PFMT sessions, emerged as significant predictors, whereas others, such as the daily duration of PFMT, did not exhibit the same significance level. These findings emphasize the intricate interplay of multiple variables contributing to the outcomes of PFMT interventions.

Existing studies have investigated predictive factors for vaginal erbium laser treatment. However, these studies excluded EFAs in their study population, despite demonstrating that age, menopausal state, body mass index, weight of newborn, initial perinometry duration, basal ICIQ scores, and total laser energy expenditure during the sessions were the significant predictive factors that could impact the success of vaginal erbium laser treatment for SUI [25,26].

Notably, our study holds direct implications for healthcare professionals managing EFAs with severe SUI. By using the identified factors, i.e., serum total testosterone levels, frequency of PFMT per week, 1HrPadtest at three months, and VEL+UEL treatment, along with their respective cutoff values, clinicians can enhance their decision-making processes and provide tailored interventions that optimize athletes' successful return to sports activities. This translation of research into practical use aims to facilitate evidence-based treatment strategies that improve these athletes’ quality of life and athletic performance.

The random forest analysis revealed important factors for predicting the return-to-sport rates in EFAs with SUI after childbirth. Among these factors, the 1HrPadtest at three months (importance: 5.945126) and frequency of PFMT per week (importance: 5.437675) emerged as highly influential factors. Total testosterone levels (importance: 4.059365) and VEL+UEL treatment (importance: 2.483492) also demonstrated some significance. These findings offer valuable insights for developing targeted interventions and treatment strategies to enhance the return-to-sport rates in this population, ultimately improving their quality of life and athletic performance.

The strength of our study lies in our ability to measure predictive factors for successful return to sports in EFAs with severe SUI for the first time. To achieve this, we leveraged artificial intelligence [27]. ChatGPT-4 proposed using appropriate statistical programming, offering the potential to enhance statistical analyses in sports medicine. However, it is important to note that evaluating clinical significance and identifying potential statistical errors in this context is complex [28].

A limitation of our retrospective study is the potential biases and limitations in data collection and analysis. The small sample size of EFAs with severe postpartum SUI may limit the generalizability of our findings. In addition, while efforts were made to address concerns related to differences in baseline characteristics between intervention groups, the study did not comprehensively assess post-operative recovery from MUS surgery and laser treatment. We primarily focused on exploring predictive factors, which prevents us from definitively elucidating the mechanism by which these factors impact SUI. Consequently, we cannot establish the clinical relevance of these correlations. When considering the improvement of SUI in EFAs who return to sports, it is challenging to draw parallels with the mechanisms underlying SUI improvement in non-athletic individuals, as no previous reports specifically address athletes. Although age, parity, and pre-existing urinary incontinence were considered in this study, the postpartum period was not specifically investigated. This study focused on EFAs with SUI lasting for at least six months or more. Therefore, it is unlikely to include women within six months postpartum. Nevertheless, recognizing the scarcity of prior research on postpartum SUI recovery is important [1,10,29,30]. The potential influence of regression toward the mean and the natural improvement of postpartum SUI over time, irrespective of PFMT, cannot be disregarded. Further prospective studies with larger sample sizes are needed to validate and strengthen our findings.

## Conclusions

Our study showcased the significance of PFMT and VEL+UEL treatments in facilitating the successful reentry of EFAs with severe postpartum SUI into sports. The 1HrPadtest conducted at three months emerged as a potent predictor of return rates, underscoring the criticality of early resolution of urinary incontinence. The frequency of PFMT per week and total testosterone levels also displayed favorable correlations with return rates. In our study, we harnessed AI for efficient and precise data analysis, augmenting our comprehension of the factors influencing return-to-sports outcomes. Four key factors were used to calculate the predicted logit(p). Furthermore, the random forest model imparted valuable insights into the relevance of these factors in forecasting return-to-sport rates. However, it is essential to recognize the constraints of our study, encompassing its retrospective design and limited sample size. To corroborate and reinforce our findings, additional prospective studies involving larger sample sizes are imperative.
